# Supplementary material for: In Silico Simulations Reveal Molecular Mechanism of Uranyl Ion Toxicity towards DNA-Binding Domain of PARP-1 Protein
Source: Biomolecules. 2023 Aug 20;13(8):1269. doi: 10.3390/biom13081269 (PMC10452222; doi:10.3390/biom13081269)
Supplement: Supplementary file 1 [file biomolecules-13-01269-s001.zip › biomolecules-2525146-SI.pdf]

SUPPLEMENTARY MATERIALS

Table S1. Detailed information on free energy profiles calculation setup. Atomic serial numbers in model structures: $OH2^{WT1}$ – 12778, $H2^{WT1}$ – 12780, U^{UO2} – 1483, SG^{Cys162} – 849 for stage I (hydrolysis); COO^{Glu190} – 1291, 1292, 1293, U^{UO2} – 1476, $OH2^{WT2}$ – 1487 for stage II (association).

Stage	*Reaction coordinate λ, Å*	*Spectrum of λ, Å*	*Number of trajectories computed*	*Harmonic force constant, kcal/mol*	*Trajectories lengths, ps*
I	d($OH2^{WT1}$, $H2^{WT1}$) + d(U^{UO2}, SG^{Cys162})	3.65 … 6.25	21	60-80	4.5-6.0
II	d(U^{UO2}, COO^{Glu190}) – d($OH2^{WT2}$, U^{UO2})	-0.80 … 2.60	32	60-80	5.0-8.0

Table S2. Bonding properties of uranyl complexes with different ligand compositions. N stands for a number of ligands of a particular type. For those formulas that have several structural representations, average values are presented.

Formula	*N(CH_3S^-)*	*N(H_2O)*	*N(OH^-)*	*Bond lengths*			*Mayer bond orders*			*IBSI*			*Charge trans. to UO_2 (-e)*		
				U-SCH_3	*U-H_2O*	*U-OH*	*U-SCH_3*	*U-H_2O*	*U-OH*	*U-SCH_3*	*U-H_2O*	*U-OH*	*SCH_3*	*H_2O*	*OH*
s2w2	2	2	0	2.67	2.48	x	0.85	0.27	x	0.26	0.21	x	0.63	0.16	x
s2w1oh1	2	1	1	2.73	2.56	2.18	0.79	0.25	0.56	0.23	0.18	0.42	0.55	0.14	0.37
s2oh2	2	0	2	2.82	x	2.22	0.65	x	0.44	0.2	x	0.38	0.47	x	0.34
s3w1	3	1	0	2.70	2.53	x	0.81	0.24	x	0.24	0.19	x	0.56	0.16	x
s3oh1	3	0	1	2.78	x	2.2	0.49	x	0.5	0.21	x	0.4	0.38	x	0.34
s2w3	2	3	0	2.71	2.59	x	0.87	0.25	x	0.24	0.17	x	0.62	0.19	x
s2w2oh1	2	2	1	2.75	2.64	2.22	0.66	0.25	0.60	0.19	0.15	0.37	0.5	0.18	0.4
s2oh3	2	0	3	3.06	x	2.29	0.44	x	0.51	0.13	x	0.35	0.25	x	0.34
s3w2	3	2	0	2.70	2.7	x	0.78	0.26	x	0.2	0.15	x	0.69	0.13	x
s3oh2	3	0	2	3.02	x	2.28	0.36	x	0.47	0.14	x	0.33	0.26	x	0.41

Table S3. Solute and solvent thermodynamic properties change upon transition from state I to state II.

Free energy component	*Value, kcal/mol*
$\Delta H_{reac}^{I\to II}$	2.8
$\Delta H_{conf}^{I\to II}$	4.4
$T\Delta S_{conf}^{I\to II}$	19.4
$\Delta H_{solv}^{I\to II}$	-4.1
$T\Delta S_{solv}^{I\to II}$	-15.7
$\boldsymbol{\Delta G_{tot}^{I\to II} = -0.6\ kcal/mol}$	

Table S4. Solute and solvent thermodynamic properties change upon transition from state I to state III.

Free energy component	*Value, kcal/mol*
$\Delta H_{reac}^{I\to III}$	-8.9
$\Delta H_{conf}^{I\to III}$	1.8
$T\Delta S_{conf}^{I\to III}$	1.1
$T\Delta S_{rot}^{I\to III}$	0.4
$T\Delta S_{trans}^{I\to III}$	< 4.2
$\Delta H_{solv}^{I\to III}$	-2.1
$T\Delta S_{solv}^{I\to III}$	-7.5
$\boldsymbol{\Delta G_{tot}^{I\to III} \sim -7.4\ kcal/mol}$	

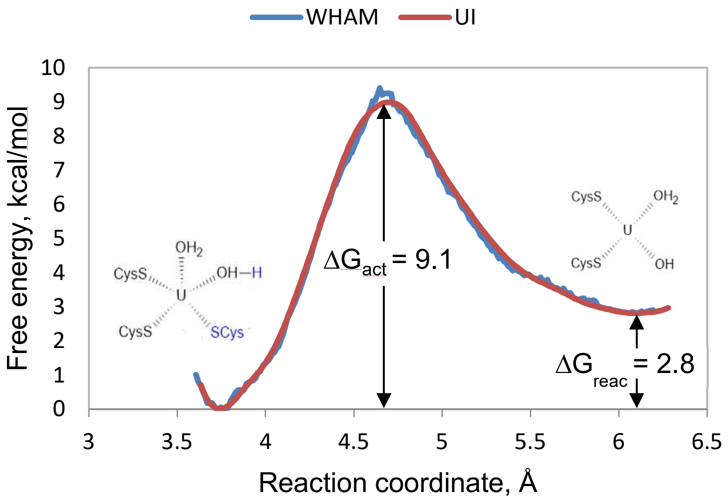

Figure S1. Internal hydrolysis reaction free energy profile derived from Umbrella sampling calculations. Color corresponds to US results analysis method. The reaction coordinate is d(OH2^{WT1}, H2^{WT1}) + d(U^{UO2}, SG^{Cys162}).

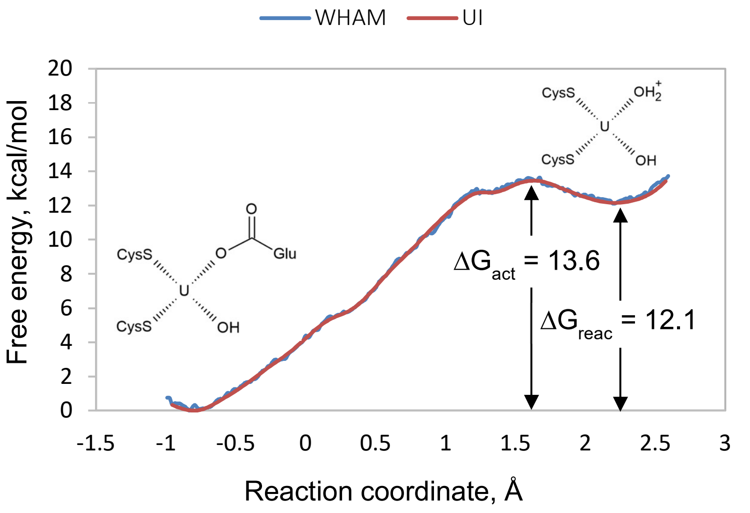

Figure S2. Association reaction free energy profile derived from Umbrella sampling calculations. Color corresponds to US results analysis method. The reaction coordinate is d(U^{UO2}, COO^{Glu190}) – d(OH2^{WT2}, U^{UO2}).
